# Optimising functional outcomes in rectal cancer surgery

**DOI:** 10.1007/s00423-020-01937-5

**Published:** 2020-07-26

**Authors:** Fabio Nocera, Fiorenzo Angehrn, Markus von Flüe, Daniel C. Steinemann

**Affiliations:** 1 Clarunis, Department of Visceral Surgery, University Centre for Gastrointestinal and Liver Disease, St Clara Hospital and University Hospital, Kleinriehenstrasse 30, 4058 Basel, Switzerland; 2 Department of Surgery, University Hospital Basel, Spitalstrasse 23, 4031 Basel, Switzerland

**Keywords:** Total mesorectal excision, Rectal cancer, Functional outcome, Health related quality of life, Pelvic floor

## Abstract

**Background:**

By improved surgical technique such as total mesorectal excision (TME), multimodal treatment and advances in imaging survival and an increased rate of sphincter preservation have been achieved in rectal cancer surgery. Minimal-invasive approaches such as laparoscopic, robotic and transanal-TME (ta-TME) enhance recovery after surgery. Nevertheless, disorders of bowel, anorectal and urogenital function are still common and need attention.

**Purpose:**

This review aims at exploring the causes of dysfunction after anterior resection (AR) and the accordingly preventive strategies. Furthermore, the indication for low AR in the light of functional outcome is discussed. The last therapeutic strategies to deal with bowel, anorectal, and urogenital disorders are depicted.

**Conclusion:**

Functional disorders after rectal cancer surgery are frequent and underestimated. More evidence is needed to define an indication for non-operative management or local excision as alternatives to AR. The decision for restorative resection should be made in consideration of the relevant risk factors for dysfunction. In the case of restoration, a side-to-end anastomosis should be the preferred anastomotic technique. Further high-evidence clinical studies are required to clarify the benefit of intraoperative neuromonitoring. While the function of ta-TME seems not to be superior to laparoscopy, case-control studies suggest the benefits of robotic TME mainly in terms of preservation of the urogenital function. Low AR syndrome is treated by stool regulation, pelvic floor therapy, and transanal irrigation. There is good evidence for sacral nerve modulation for incontinence after low AR.

## Introduction

By the introduction of total mesorectal excision (TME) [1], neoadjuvant chemoradiation (CR) [2, 3], improved accuracy of preoperative imaging [4] and better quality assessment of surgical specimens [5], the oncological outcome of rectal cancer surgery has strongly improved. Within a multimodal treatment, a distal resection margin of ≥ 1 mm may be considered adequate and thus does allow restorative resection even in very low rectal cancer [6–8]. Moreover, several large-scale randomized clinical trials (RCT) have demonstrated the non-inferiority regarding the oncological outcome of laparoscopic surgery compared with open surgery for rectal cancer [9–12]. Laparoscopic surgery enables faster recovery with reduced morbidity, reduced surgical site infections, less pain and a shorter hospital stay compared to open surgery [13–16].

Functional outcome and health-related quality of life (HRQOL) after rectal cancer surgery become of ever-increasing importance considering improvements in survival. Multimodal treatment of rectal cancer is still associated with an inherent risk of important functionality changes to bowel, anorectal and urinary, as well as sexual function. The incidence of low anterior resection syndrome (LARS) is estimated between 37 and 90% after rectal resection [17]. Deterioration of function has an important impact on HRQOL.

The goal of this review is to compile different functional changes after rectal cancer treatment. Furthermore, preventive strategies as well as therapeutic options to address the functional deterioration in each domain will be described.

### Anorectal and bowel function

Normal defaecation involves a well-coordinated sequence of events at a semi-voluntary level. Smooth and striated muscles, as well as the central, somatic, autonomic and enteric nervous system are required. The rectum serves as a storage reservoir and as a pump for evacuation of faeces. Additionally, the anal canal and the surrounding pelvic floor play an important role during defaecation [18, 19]. Before defaecation the rectum is mostly empty. Defaecation is initiated by rectal filling and distension. Conscious awareness results in an urge to defaecate as a distension threshold is reached. The contraction of the rectum is followed by relaxation of the internal anal sphincter (IAS) using the recto-anal inhibitory reflex (RAIR). Simultaneously, the external anal sphincter (EAS) is activated to allow rectal contents to move in the upper anal canal to determine the nature of the content. When defaecation is voluntarily allowed the tonic activity of the pelvic floor is inhibited and the puborectal sling is relaxed. EAS is relaxed and by activation of the longitudinal muscles the cushions of the anal canal are flattened. All this is necessary to allow the intrarectal pressure to surpass the pressure of the anal canal. There is a predefaecatory increase in propagatory sequences of motor activity in the rectum. Normal colonic motility includes segmental activity in order to move the faeces slowly distally towards the rectum. There are low amplitude propagated contractions (LAPCs) and high amplitude propagated contractions (HAPCs). HAPC has the function to transport fluid content while LAPC is associated with distension of the viscus and passage of flatus. Furthermore, HAPC plays an important role during defaecation itself. Sleep inhibits colonic motor activity while ingestion of food is a major stimulus [20]. HAPC may precede defaecation but not every HAPC is followed by voluntary defaecation. It has been demonstrated that distension of the sigmoid colon does result in a pressure increase in the rectosigmoid junction limiting the rectal filling [21].

**Fig. 1:**
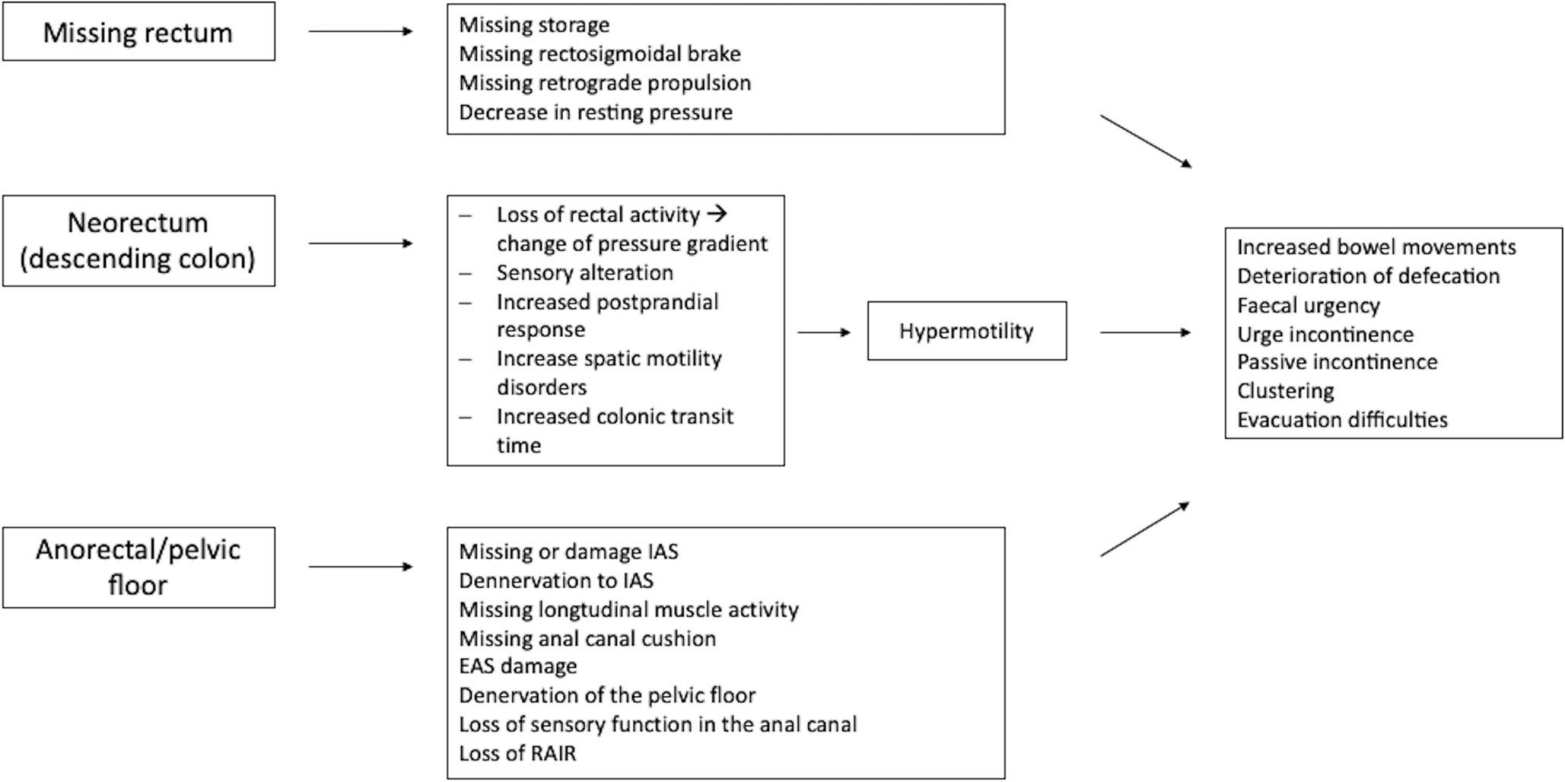
Injury levels, anorectal and bowel dysfunctions and subsequent symptoms after anterior resection (AR)

In low anterior resection (LAR) injury causing anorectal and bowel malfunction may occur at different levels (Fig. 1).

#### Missing rectum

It is intuitive that rectal dissection results in the loss of a reservoir for stool. Accordingly, a low level of the anastomosis has been identified as a risk factor for the development of LARS in a multi-centre study including 578 patients [22]. Hence, the odds ratio for LARS of TME versus partial mesorectal excision (PME) is 2.81 [23]. Neoadjuvant radiotherapy does further deteriorate the function of the rectal remnant. In a manometry study, 1 year after either LAR alone or CR and LAR not only a decrease in the resting pressure in the latter group was noticed but also a lower rectal compliance [24]. Consistently in a follow-up study, 2 years after external beam radiotherapy for prostate cancer a decrease in rectal capacity and a deterioration of sensory function was found [25].

For the reconstruction of the missing rectum, the mobilized descending colon is pulled down into the pelvis. The colon replacing the rectum is called the “neorectum”. Unfortunately, the neorectum does not behave like the rectum. Ziv postulated that loss of rectal activity is associated with the decrease of the anal-resting pressure. This leads to a change in the pressure gradient between the neorectum and the anal canal. These changes are responsible for faecal soiling [26]. There is evidence for some sensory adaptation within neo-rectal reservoirs. However, poorly compliant neorectum show sensory alteration correlating with incontinence more commonly in patients with preexisting sphincter damage [26, 27]. Koda measured the intracolonic pressure after LAR with a high tie versus a low tie. They could demonstrate a lack of propagating contractions and an increase of spastic motility disorders in the high tie group as well as increased colonic transit time [28]. Spastic hypermotility of the neorectum has been confirmed by others [29]. As a sequela of neuronal damage to the neorectum, an increased postprandial response with high pressure within the neorectum has been observed [30]. Innervation may also be damaged while rectal mobilization or following surgery when inflammation and fibrosis take place resulting in intramural nerve plexus damage [31–34].

#### Damage to the anus and pelvic floor

Neurological or structural damage to the IAS leads to passive incontinence (unconscious leakage), whereas injury to the EAS usually results in faecal urgency. IAS damage occurs in up to 18% due to direct injury by endoanal instrumentation such as the introduction of the stapler device [26, 35]. Even in sigmoid resection with stapled anastomosis, a temporary deterioration of the IAS function has been reported [36, 37]. IAS damage may also result from inter-sphincteric resection (ISR) [35, 38].

Moreover, damage to the nerval supply to the IAS occurs from injury to the sympathetic and parasympathetic nerve fibres on the posterolateral side of the prostate [39]. The study by Koda showed that a decrease in the anal canal high-pressure zone will lead to severe postoperative defaecatory malfunction [40].

The anorectum is attached to the muscles of the pelvic floor by the conjoint longitudinal muscle. It is activated during defaecation and its contraction induces the shorting of the anal canal. In case of LAR and especially ISR, the anorectum is detached from the pelvic floor thus leading to deterioration of defaecation [41, 42].

#### Incidence and assessment of low anterior resection syndrome

Major LARS occurs in 37 to 90% of patients after LAR [43–47]. Severe incontinence is prevalent in ultralow anterior resection (AR) in 49% and in ISR even in 76% [48]. Previously the symptoms were thought to be transient, mainly resolving by 12 months after AR [49]. However, several long-term studies are now reporting the presence of symptoms up to 15 years after AR, with the prevalence of faecal incontinence varying from 0 to 71% and rectal evacuation disorders from 12 to 74% [50, 51]. Furthermore, a recent meta-analysis showed that the estimated long-term prevalence of majors LARS after rectal cancer surgery was 41% [52]. These results indicate that this syndrome is not a transient irritability of the neorectum in the postoperative period, but a result of permanent changes [17].

The LARS score is a valid and reliable score correlated to HRQOL. The range is divided into 0 to 20 (no LARS), 21 to 29 (minor LARS) and 30 to 42 (major LARS) [53]. Patients with LARS fall into two groups: those with urgency or faecal incontinence and those with evacuation dysfunction, although symptoms often overlap [17].

However, LARS is not specific for AR. Major LARS in the age group 50 to 79 years are reported in 10 to 19% of the general population [54, 55]. Furthermore, in a Swedish prospective study, major LARS was found in 20% after right colonic resection and 16% after left colonic resection [56].

The Rockwood scale, the St. Mark’s incontinence score, the Memorial Sloan-Kettering Cancer Centre Bowel Function Instrument system (MSKCC BFI), the Wexner score and the abovementioned LARS score are the most commonly used tools assessing faecal incontinence [53, 57–61].

### Prevention of low anterior resection syndrome

#### Non-operative management

Considering the important changes in bowel function and the associated deterioration of HRQOL the indication for LAR is questioned in patients with early rectal cancer as well as patients with clinical complete remission after neoadjuvant CR [62].

After neoadjuvant CR in 8 to 27%, a complete pathological response is achieved [63–65]. An alternative approach in patients with a complete response is non-operative management (NOM). Most patients with NOM may avoid surgery and a definitive colostomy with reasonable anorectal and urinary function [66]. The matched controlled study by Hupkens et al. showed a better HRQOL, better physical and emotional rates and better global health status after NOM compared to LAR. These patients had also fewer problems with defaecation and sexual and urinary tract function. However, CR therapy on its own is not without long-term side effects. One-third of the NOM patients experience major LARS symptoms, as compared with 67% of the patients after TME [67]. The meta-analysis of Dossa et al. including 867 patients demonstrated that most patients within NOM avoid major surgery with a similar survival rate compared with LAR. Nevertheless, there is a lack of a high level of evidence studies [68]. NOM is associated with an inherent higher local recurrence rate but not a worse overall survival. Given the very low level of evidence, NOM should only be considered within studies [69].

Local excision by transanal endoscopic microsurgery (TEM) should be considered in low-risk T1 cancers [70]. In the CARTS study, 55 patients with a median age of 64 years with T1-3 N0 (EMVI-, G1-2, V0, L0) rectal cancer were evaluated for CR followed by TEM. Of those, 85% underwent TEM after achieved downsizing. Four patients (9%) developed local recurrence within 12 months and underwent salvage TME. The 5-year disease-free survival was 81.6%. However, three of four patients with local recurrence also developed distant metastasis and died. Moreover, another 4 patients had metachronous distant metastasis. Of the patients undergoing organ-preserving therapy 50% had major LARS 48 to 68 months after treatment [71]. In the GRECCAR 2 trial 148 patients with cT2-3 low rectal cancer and downsizing after chemoradiation (residual tumour < 2 cm) were randomly assigned to TEM or TME. Completion TME was performed in 26 patients of the TEM group in the case of ypT2-3. The two-year local recurrence rate was 5% in the TEM and 6% in the TME group (*p* = 0.68) and the disease-free survival 78% and 76% (*p* = 0.45). The incidence of faecal incontinence was 5% and 14% (*p* = 0.34) and the rate of sexual dysfunction 23% and 18% (*p* = 0.81) [72]. The failure to demonstrate a superior functional outcome in the TEM group was explained by the high rate of completion TME. The ongoing GRECCAR 12 trial will investigate the outcome with more restrictive indications for completion TME (only ypT3 or ypT3cN+ or R1) and with the inclusion of neoadjuvant induction chemotherapy. Currently, local excision in > pT1 low-risk cancer should only be considered within clinical trials or in frail patients.

#### No radiotherapy

The high level of anorectal and bowel dysfunction in NOM demonstrates that CR alone contributes importantly to LARS. [57, 73–75]. The study by Lange et al. showed that 5 years after surgery faecal incontinence occurred in 62% of patients who had preoperative radiotherapy and only in 39% of patients who had not [76]. Another recent study performed in England showed that patients who received preoperative radiation therapy had higher odds of reporting disturbed bowel control (OR 1.55), severe urinary leakage (OR 1.69) and sexual difficulties (OR 1.73) compared with those who had surgery alone. In addition, patients who received long-course CR reported better bowel control than those who had short-course radiation therapy [77]. The 15-year’s results of an RCT of preoperative short-course radiotherapy followed by TME versus TME alone showed worse functional results for the first group in terms of faecal incontinence (12/21 vs. 11/42, *p* = 0.01) and urinary incontinence (45% vs 27%; *p* = 0.02) [78]. The use of pads for faecal incontinence is increased for preoperative CR vs TME alone 5 years after treatment (51.5% vs. 30.5%) [79]. Neoadjuvant CR is beneficial and recommended in stage II and III rectal cancer [63]. However, in cT3a and cT3b (invasion of the mesorectum < 5 mm) cN0, EMVI-cancers the omission of neoadjuvant radiation may be considered assuming a local recurrence rate similar to stage I cancer in this specific subset of patients [80, 81].

#### No restoration

No difference in the general HRQOL comparing AR and abdominoperineal resection (APR) was found in a meta-analysis including 1443 patients [82]. The decision whether to perform APR or AR should—beside oncological considerations—include an assessment of the anticipated bowel function. The preoperative LARS score (POLARS) is a nomogram that allows the estimation of postoperative bowel function. Predictive factors included in the POLARS score are tumour height, age, TME vs. PME, protective defunctioning stoma and preoperative radiotherapy [83]. Further factors to be considered in the decision-making should be co-morbidities, preoperative continence and sphincter function as well as cognition, coping, lifestyle and expectations of the patient. In a study, patients were asked prior to surgery for their choice. While 30% opted for AR only 5% decided for APR but 65% chose to leave the decision to the surgeon. Four years after, surgery patients were asked again how they would choose if they could decide again. Of the APR patients, 46% would choose again for APR, but 22% for AR, 32% would leave the decision to the surgeon. Of the AR patients, 69% would again decide for AR and only 4% for APR. The remaining 28% leave the decision to the surgeon. The sequela of AR seems to be more acceptable than those of APR [84]. These findings underline the importance to involve the patient in this important decision prior to surgery.

#### Partial versus total mesorectal excision

As previously discussed, the height of anastomosis plays an important role in the risk of developing LARS. Coloanal anastomosis showed the absence of recto-anal inhibitory reflex (RAIR), which is replaced by a sharp contraction, and a lower anal resting pressure when compared with controls. The absence of RAIR is the consequence of the complete excision of the rectum such as in ultralow AR with coloanal anastomosis. RAIR recovers in most cases by the end of the second postoperative year, but the coordinated sensory-motor integration of the rectum remains distorted. Conclusively, the lower the resection is performed, the more functional complications may occur [28, 30, 34, 85, 86].

TME is a risk factor to develop LARS. Bregendahl et al. who analysed functional outcomes after curative resection for rectal cancer showed a higher risk of developing a major LARS after TME as compared to PME (OR = 0.21) [57]. In tumours of the upper rectum, a distal resection margin of 5 cm is sufficient. Within the mesorectum, tumour spreads are found up to 40 mm distal from the tumour. In tumours located more than 10 cm from anal verge, a PME should be performed to avoid unnecessary deterioration of bowel function.

#### Technique of anastomosis

Reduced neorectal reservoir volume, resulting from the construction of a conventional end-to-end anastomosis is presumed to be the cause of urgency and incontinence and has led to develop alternative configurations. Colonic J-pouch, transverse colosplasty and side-to-end anastomosis have a similar outcome in terms of frequency, urgency, continence, evacuation function and HRQOL (Table 1). For the ease of construction, side-to-end anastomosis should be the preferred reconstruction.

**Table 1:** Studies evaluating the technique of anastomosis in anterior resection (AR) for rectal cancer surgery

Recommendation/results	Design	Evidence*	Reference
Reduced defaecation frequency and urgency in J-pouch and transverse coloplasty compared to straight anastomosis	MA	2a	[87]
Fewer evacuation disorders after transverse coloplasty compared to J-pouch	RCT	1b	[88–90]
J-pouch has similar surgical and functional results as side-to-end anastomosis whereas transverse coloplasty has no advantage	RCT SR	1b 2a	[91–93] [94]
J-pouch, side-to-end anastomosis and transverse coloplasty lead to better functional outcomes compared to straight anastomosis	MA	2a	[95]
Comparable HRQOL, functional outcomes and complications rates one and two years after J-pouch or side-to-end anastomosis	RCT	1b	[96]
No difference in terms of evacuation and incontinence scores 6, 18 and 24 months postoperatively comparing side-to-end anastomosis, straight anastomosis and J-pouch	RCT	1b	[97]
Incidence of severe or moderate incontinence 36 months after ultralow AR; hand-sewn 94.2% vs. double stapling 38.8%	POS	3b	[98]
No difference in evacuation function 12 months after surgery in J-pouch vs. side-to-end anastomosis	RCT	1b	[31]

*MA*, meta-analysis; *RCT*, randomized controlled trial; *SR*, systematic review; *POS*, prospective observational study

*Level of evidence (March 2009)—Oxford Centre for Evidence-based Medicine

Regarding the performance of anastomosis, a meta-analysis analysing functional outcomes of stapled versus hand-sewn ileal pouch-anal anastomosis (IPAA) following proctocolectomy in ulcerative colitis showed that stapled IPAA offered improved nocturnal continence [99]. Similarly, the incidence of severe or moderate incontinence 36 months after ultralow AR with hand-sewn coloanal anastomosis versus double stapling LAR was 94.2% and 38.8% in a large observational study [98].

#### Surgical technique (laparoscopy, robotic, taTME)

It has been questioned whether anorectal function might be worse in patients who received a temporary stoma in LAR. In a Swedish RCT 234, patients were randomly assigned to receive a defunctioning stoma or not for LAR with an anastomosis < 7 cm from the anal verge. The rate of symptomatic anastomotic insufficiency did largely differ (10.3% vs 28%, *p* < 0.0001) [100]. In the 12-year follow-up 46 with and 41 without initial temporary stoma were available for a functional follow-up. The rate of patients experiencing a symptomatic anastomotic insufficiency did not differ in the groups of followed patients. Increased rates of incontinence for flatus (*p* = 0.03), and liquid stool (*p* = 0.005) but not for major LARS were found in the stoma group [46]. However, it should be noted that in the initial trial more patients in the stoma group had preoperative radiotherapy [100]. This difference was no longer significant in the 12-year follow-up (93% in the stoma group, 80% in the no stoma group); but the sample size might be too small to detect a difference [46]. In a retrospective analysis of 150 LAR patients, the multivariate analysis showed that the creation of protective stoma and the time to ileostomy closure were not risk factors for LARS [101]. However, in an RCT comparing early (8–13 d) and late (> 12 weeks) ileostomy closure no difference in major LARS (29/40 and 25/42, *p* = 0.25) was found but worse scores for soiling were detected in the late closure group (*p* = 0.017) [102].

Regarding HRQOL, RCTs showed conflicting results comparing laparoscopic and open surgery. There is a lack of studies comparing laparoscopic and open LAR concerning the functional outcome. A few case-control studies compare transanal TME (ta-TME) and laparoscopic TME. They demonstrate comparable outcomes or even worse functional results for ta-TME. In case-control studies, robotic LAR was found to be beneficial in terms of urogenital function preservation. However, no difference for LARS was demonstrated (Table 2).

**Table 2:** Studies evaluating the open, laparoscopic, robotic and transanal surgical technique for rectal cancer

Recommendation/results	Design	Evidence*	Reference
Laparoscopic vs. open technique			
Better scores in HRQOL and less defaecation problems in laparoscopic vs. open AR	RCT	1b	[103]
No differences in HRQOL in 12-month follow-up laparoscopic vs open AR	RCT	1b	[104]
Transanal TME (taTME) vs. laparoscopic TME			
Better oncological and functional results, comparable pathological results, acceptable short-term postoperative outcomes, shorter operation time, less blood loss and shorter hospital stay after taTME vs laparoscopic	CS MA	3b 3a	[105–107] [108]
Comparable functional outcomes taTME comparing with laparoscopy	CS MA	3b 3a	[109–111] [112]
Higher anorectal dysfunction in taTME compared to laparoscopic including buttock pain, diarrhoea, clustering of stools and urgency	CS	3b	[113]
Robotic vs. laparoscopic technique			
Earlier recovery of voiding and sexual function after robotic TME vs laparoscopic	CS	3b	[114–116]
Robot-assisted surgery may be technically more efficient, especially in low-lying tumours requiring inter-sphincteric resection and complex pelvic dissection	CS	4	[117]
Robotic and laparoscopic inter-sphincteric resection show comparable results Benefits of robotic ISR should be evaluated in larger RCTs	MA	3a	[118]
No difference in major LARS between laparoscopic and robotic TME	RCT	1b	[119]

*MA*, meta-analysis; *RCT*, randomized controlled trial; *POS*, prospective observational study; *ES*, evaluation study; *CS*, comparative study

*Level of evidence (March 2009)—Oxford Centre for Evidence-based Medicine

#### Intraoperative neuromonitoring

A prophylactic approach to avoid nerve damage and the associated deterioration of functional outcomes is intraoperative neuromonitoring (IONM). Kneist et al. conducted a prospective study with a small group of patients undergoing LAR with IONM through pelvic splanchnic nerve stimulation under continuous electromyography of the IAS. The study showed that all patients with positive IONM signals were continent after stoma closure [120]. A consecutive case-control series by the same researchers demonstrated a significant lower rate of urinary and anorectal dysfunction after using IONM [121]. In addition, the laparoscopic neuromapping seems to be an appropriate method for reliable quality assurance of laparoscopic nerve-sparing surgery [122]. Kauff et al. compared pelvic IONM during TME with a control group and demonstrated the less new onset of faecal incontinence in the neuromonitoring group at each follow-up (3, 6. 12 and 24 months) [123]. Zhou et al. demonstrated no difference between preoperative and postoperative urogenital and anorectal function in patients with positive IONM. Those patients exhibited higher International Prostate Symptom Score, a lower IIEF-F and a lower Female Sexual Function Index score 12 months postoperative compared with patients with negative IONM [124]. The ongoing NEUROS RCT by Kauff et al. will hopefully provide high-quality evidence on the efficacy of pelvic IONM aiming for the improvement of functional outcome in rectal cancer patients undergoing TME [125].

### Therapy of anorectal and bowel disorders

#### Pharmacological therapy

There are a few studies regarding treatment for LARS. For diarrhoea-predominant LARS with incontinence for liquid stool and in case of increased frequency, loperamide is used. Loperamide increases sphincter resting pressure by 20%. Reduced bowel frequency and improved nighttime continence are reported [126]. Good results are experienced with managing incontinence and clustering with psyllium. In a cross-over study in incontinent patients, both loperamide and psyllium were equally effective, whereas less unwanted constipation occurred with psyllium [127].

Itagaki et al. showed the effectiveness of serotonin receptor antagonists (5-HT3) for the treatment of LARS, as in diarrhoea-predominant irritable bowel syndrome. Functional outcomes improved after taking ramosetron for 1 month [128]. 5-HT3 are effective because of their ability to slow gut transit. Especially in patients with postprandial urgency due to increased propagatory propulsions in the neorectum 5-TH3 antagonists have been shown to be effective. [129].

Increased flatulence and bloating may be associated with small intestinal bacterial growth (SIBO). SIBO is treated with antibiotics such as rifaximin and neomycin [130]. Stephens et al. failed to demonstrate any difference in bowel function in patients randomly assigned to placebo or probiotics after the reversal of a temporary loop ileostomy as a result of prior rectal resection [131].

#### Pelvic floor rehabilitation

Pelvic floor rehabilitation (PFR) includes pelvic floor muscle training (PFMT), biofeedback (BF) and rectal balloon training (RBT) and is accepted treatment for faecal incontinence. PFMT improves the structural support, the timing and strength of automatic contractions resulting in reduced leakage. BF uses visual and hearing signals to inform patients about internal physiological events. This therapy leads to a more precise discrimination of rectal sensation and synchronise voluntary contraction of the EAS as a response to rectal sensation. The RBT has the aim to improve rectal sensitivity by stepwise reductions in rectal balloon distension, in order to learn to distinguish smaller rectal volumes. In case to resist urgency, a progressive distension is performed or it can be used to counteract the RAIR by using the voluntary anal squeeze [132]. A systemic review, an RCT, and a few case-control studies demonstrated a reduction in incontinence scores, reduced stool frequency, and improved HRQOL (Table 3).

**Table 3:** Studies evaluating pelvic floor rehabilitation (PFR) for low anterior resection syndrome (LARS)

Recommendation/results	Design	Evidence*	Reference
Incontinence score was improved after pelvic floor rehabilitation	SR	2a	[133]
Lower mean stool frequency in patients after sphincter training compared without training Both groups similar continence score (Wexner score 8.3 vs. 9.9) Less dyschezia and improved HRQOL after training	CS	4	[134]
Positive short- and long-term effects of pelvic floor rehabilitation and biofeedback training in patients with faecal incontinence after surgery plus CR and in patients with surgery alone Increase in modified Cleveland Incontinence Score	CS	2b	[135]
Improvement of Wexner score, number of bowel movements and anorectal manometry after biofeedback training in patients with LARS	CS	4	[136]
Rectal balloon training with pelvic floor muscle training is equally effective as pelvic floor muscle training alone Beneficial effect of rectal balloon training on urgency control, global perceived effect and lifestyle adaptations	RCT	1b	[132]

*SR*, systematic review; *RCT*, randomized controlled trial; *CS*, comparative study

*Level of evidence (March 2009)—Oxford Centre for Evidence-based Medicine

#### Transanal irrigation

In a subgroup of patients with LARS, a positive effect of transanal irrigation (TAI) was noted (Table 4). However, only a third of patients with TAI are willing to continue this therapy [139, 140]. During TAI the LARS score dropped from 35 to 12 after 6 months but rose to 27 3 months after the suspension of TAI in a study of Martelucci et al. [142]. When TAI is discontinued there seems to be no sustained improvement in LARS.

**Table 4:** Studies evaluating transanal irrigation (TAI) for low anterior resection syndrome (LARS)

Recommendation/results	Design	Evidence*	Reference
Fewer complaints of constipation, less faecal incontinence, improved HRQOL after TAI compared to best supportive care	CS	4	[137, 138]
Effect of 79% to 100% after TAI in patients with defaecation disturbances after pouch surgery or LAR 1/3 of the patients are willing to continue Perforation rate of sigmoid colon or rectum is 0.002%	SR	3a	[139, 140]
Decrease of number of defaecations during the day and night, Cleveland incontinence score decreased, the mental component of SF36 and all domains of the Rockwood QoL instrument improved	CS	4	[141]
Decrease number of median daily bowel movements after TAI LARS score dropped from 35.1 to 12.2 after 6 months but rose to 27 3 months Four components of the SF-36 improved during the TAI period and the MSKCC BDI score significantly improved in several domains	CS	4	[142]

*SR*, systematic review; *CS*, comparative study

*Level of evidence (March 2009)—Oxford Centre for Evidence-based Medicine

#### Sacral neuromodulation

Sacral nerve modulation (SNM) has proven to be effective in several small studies [143–151]. A meta-analysis showed a reduction of the frequency of weekly episodes of incontinence and an improvement of the ability to defer defaecation [150]. After device implantation, the mean incontinence score decreased, and the mean number of incontinence episodes dropped. Manometric parameters were consistent with clinical results: maximum and mean resting tone and the squeeze pressure were normal in the patients with improved incontinence symptoms [143]. Miguel et al. demonstrated a reduction in the Cleveland Clinic Florida Faecal Incontinence scoring system (CCF-FI) after device implantation as compared to preimplantation [144]. Ramage et al. reviewed the literature in 2015 [149]. Of the patients, 79.1% proceeded to permanent SNM implantation. In five studies, the main factor to proceed with permanent implementation was a peripheral nerve evaluation threshold of more than 50 to 70% symptom improvements [143–145, 147, 151]. Furthermore, reduction of nocturnal defaecation, fragmentation, urgency and soiling in two-thirds of the patients after implantation of SNM were reported [148]. The mean Wexner score was significantly reduced from 17.7 to 4.6 and the mean LARS score from 36.9 to 11.4 [149]. Interestingly overall efficacy of SNM is comparable with results found after SNM for other causes of faecal incontinence. SNM has been shown to be effective in LAR patients after neoadjuvant CR with a sustained reduction of incontinence scores and an increase in HRQOL [148]. In another retrospective series of incontinence predominant LARS patients, the permanent implantation rate of SNM was 70% with an improvement in incontinence scores up to 5 years after implantation [152]. In a systematic review of Ram et al., 114 patients with SNM in the context of LARS were identified. The overall success rate was 83% with sustained improvement of HRQOL [153]. Huang et al. found a mean reduction in LARS of 17.8 points by SNM in their meta-analysis [154]. D’Hondt et al. analysed 11 patients undergoing SNM for LARS and found an amelioration not only in incontinence but also reduced clustering, reduced bowel movements and reduced urgency [155].

Another therapeutic approach is tibial nerve stimulation, which could be divided into two forms; percutaneous (PTNS) and transcutaneous (TTNS). PTNS and TTNS seem to improve in some outcome measures, but TTNS was not superior to sham stimulation in a large powered RCT [156]. PTNS consists of the insertion of two small electrodes above the medial malleolus adjacent to the posterior tibial nerve and another placed under the arch of the foot. Both electrodes are connected to the neurostimulator in general for 30 min in one procedure. The CONFIDENT-study as a large double-blind, multicentre RCT found no significant benefit of PTNS over sham electrical stimulation [157].

### Urogenital function

Sympathetic and parasympathetic nerves of the superior and inferior hypogastric plexus control bladder and sexual function. Sympathetic nerves are responsible for male and probably female ejaculation, while parasympathetic nerves cause erection, lubrication and swelling of the labia and the clitoris [158]. Sexual disorders may occur postoperatively for multiple reasons [159]. Among women sexual dysfunction may occur due to fatigue, depression, loss of independence and changes in relationships [160]. For men, there is also a relation to age [161]. In male patients, a diminished erection and ejaculation may be due to damage to the inferior hypogastric plexus on one side. Damage on both sides leads to impotence and bladder denervation. Retrograde ejaculation occurs when superior hypogastric plexus and/or the hypogastric nerve on both sides are damaged [162–164], whereas damage to both sides of the inferior hypogastric plexus results in female dyspareunia, decreased ability to reach orgasm, arousal and diminished vaginal lubrication may arise by unilateral injury of the inferior hypogastric plexus [163]. The main cause of postoperative sexual dysfunction is an intraoperative injury to the neurovascular bundles [165]. Leaving the Denonvilliers’ fascia intact on the prostate side during the anterior rectal dissection is mandatory to preserve these autonomic fibres. An exception to this rule are advanced tumours located on the anterior aspect of the rectum [166].

Focusing on the bladder function parasympathetic nerves control the detrusor muscle, the relaxation of the urethra and inhibit the nerve activity of the external urethral sphincter, while the sympathetic nerves control the urinary continence [158, 167]. Direct nerve damage happens during the mobilisation or traction of the rectum and might be an explanation for the improvement of the voiding dysfunction in many patients months after operation [167, 168]. Inflammation in the perivesical tissues, altered anatomy, immobilisation, failure of perineal relaxation caused by pain, failure to open the bladder neck due to stress-induced sympathetic over-activity, bladder distension and reduced contractility could all be additional indirect causes of urinary dysfunction [167]. Furthermore, adjuvant radiation therapy causes fibrosis of the bladder and urethral sphincters, which negatively affects the bladder function [169, 170].

The most common symptom in bladder dysfunction after rectal cancer surgery besides stress incontinence and urgency is the difficulty of emptying the bladder. Damage to inferior hypogastric nerve plexus leads to emptying failure, especially when performed on both sides [162–164]. Early postoperative urinary catheter removal decreases urinary tract infections rate, accelerates patient mobilisation and decreases length of stay. Nevertheless, the exact timing of the removal remains unclear [171, 172]. There is one ongoing RCT aiming to determine the optimal time slot for urinary catheter removal after laparoscopic AR [173]. Lange et al. showed an improvement of emptying dysfunction after 3 months, whereas symptoms that last up to 6 months seem to be permanent. Long-term dysfunction is reported by 31% of patients [167].

#### Incidence and assessment of urogenital dysfunction

A high percentage of patients experience new sexual dysfunction and discontinue sexual activity posttreatment [174–176]. A large number of studies have focused on bowel dysfunction only. In comparison, there is far less information about urinary and sexual dysfunction. More than half of the patients experience a deterioration in sexual function, consisting of ejaculatory problems and impotence in men and vaginal dryness and dyspareunia in women [177, 178].

Urinary dysfunction occurs in one-third of patients treated for rectal cancer due to surgical nerve damage [158, 177]. Toritani et al. observed urinary dysfunction in 8.8% of the patients after autonomic nerve-preserving surgery for rectal cancer. A multivariate analysis showed that tumour location in the lower rectum, tumour diameter > 39 mm, operation time > 239 min, blood loss > 299 ml and diabetes were independent risk factors of urinary dysfunction [179]. The incidence of postoperative urinary retention is reported in up to 18.5%. [180].

The International Prostate Symptom Score (IPSS) and Bristol female lower urinary tract symptom (BFLUTS) questionnaire are the most internationally validated scores and have been used most widely for assessing urinary dysfunction in male and women [181, 182]. The International Index of Erectile Function (IIEF) and the Female Sexual Function Index (FSFI) questionnaire are the most widely used and internationally recognised and validated tools for assessing male and female sexual dysfunction [183, 184]. Other self-made questionnaires should no longer be used. The IIEF is limited by the superficial assessment of the psychosexual background and assessment of the partner relationship, both important factors for sexual dysfunction. Another recently validated score is the Sexual Functional Vaginal Changes questionnaire. The score includes 7 items with a range of 0 to 29 points. A score > 8 indicates a sexual dysfunction and has a sensitivity of 76% and specificity of 75% detecting patients bothered by sexual dysfunction with a negative impact on HRQOL [185].

#### Prevention of dysfunction

Effective therapeutic modalities for patients with urinary dysfunction after rectal cancer treatment are missing. The best therapy remains prevention. The identification and preservation of pelvic nerves is of utmost importance. IONM showed improved functional results in some studies [120–125, 186]. IONM exhibited a higher International Prostate Symptom Score, a lower IIEF-F and a lower Female Sexual Function Index score at 12 months postoperatively [124].

In the HIGHLOW trial, 214 patients were randomised to a high or low tie of the inferior mesenteric artery in laparoscopic LAR. Patients in the low tie group reported better urinary continence, less obstructive urinary symptoms and better sexual functioning 9 months after surgery [187].

Laparoscopic surgery gained acceptance for better functional outcomes. Surprisingly, the first laparoscopic results have shown a higher prevalence of male sexual dysfunction, but not in urinary disorders compared to open surgery. Later better results were reported [188–192]. Another RCT showed no difference in sexual dysfunction and micturition symptoms after laparoscopic versus open AR [193].

After robotic TME earlier recovery of voiding and sexual function due to more precise dissection compared to laparoscopy has been demonstrated [114, 115, 194]. A recently published retrospective cohort by Yamaoka showed an inversely correlation of robotic AR with postoperative early urinary dysfunction [195]. Moreover, the systematic review by Broholm et al. presented improved urogenital function results after robotic AR compared to laparoscopy [196]. So did another recent meta-analysis, which showed better bladder function after 12 months and better sexual function after 3 months in the robotic group compared with laparoscopy [197]. Improved sexual functioning has been observed in an RCT comparing robotic and laparoscopic TME (*p* = 0.032) [198]. On the other hand, Celentano et al. state that there is no evidence to date in favor of any surgical approach concerning sexual results [199].

#### Therapy of urogenital disorders

Partial nerve damage occurs often but may improve and resolve 6 months postoperatively. Erectile dysfunction has been noted to return within 6–12 months after nerve injury. However, therapy should be started as early as possible because delayed treatment may lead to permanent dysfunction [200]. Psychotherapeutic help is important regarding psychological, emotional or social factors. It helps to regain the corporal image. Another therapeutic approach is a medication with sildenafil. Nishizawa et al. showed a satisfactory improvement of 69% of male patients with erectile dysfunction after LAR [201]. As an alternative, urdenafil with 85% satisfactory improvement was described [202]. Only after drug therapy failed, physical treatments, either with intracavernosal injection or implanted silicone rods, inflatable penile implants and vacuum devices are available [201–203]. In women, management is based on sex therapy and psychotherapy, especially for libido disorders. For vaginal atrophy and dryness topical oestrogen is recommended [204].

Diversionary or occlusive devices and absorptive incontinence pads or undergarments are available for long-term urinary incontinence. Patients with compliance abnormalities (failure to store) may be treated with a combination of medication and surgery. Anticholinergic medications may decrease the pressure within the bladder during storage as well as marginally increase bladder volumes. As an alternative, the use of SNM for the refractory overactive bladder may be considered [203]. Furthermore, repeated injection of botulinum toxin in the detrusor muscles in patients with refractory detrusor over-activity show acceptable results [205, 206].

## Conclusion

In conclusion, several considerations are to be made to prevent dysfunction after rectal cancer surgery. The indication for TME should be decided at a multidisciplinary tumour conference and well balanced against NOM or local excision. Further multicentre RCTs are needed in order to define the indications for neoadjuvant radiotherapy more accurate as CR largely contributes to LARS. For tumours of the upper rectum, PME should be preferred. If possible, a low tie of the inferior mesenteric artery should be performed. The decision for restoration versus APR should be based on a formal assessment including POLARS and all relevant factors must be included. If restoration is decided, side-to-end anastomosis in a double-stapling technique seems to be the best anastomotic technique. The performance of an ileostomy does not increase the risk for later LARS, but the role of timing of stoma closure is still unclear. Further high-evidence clinical studies are required to clarify the benefit of IONM and to assess the functional outcome of robotic versus laparoscopic AR and ta-TME.

**Fig. 2:**
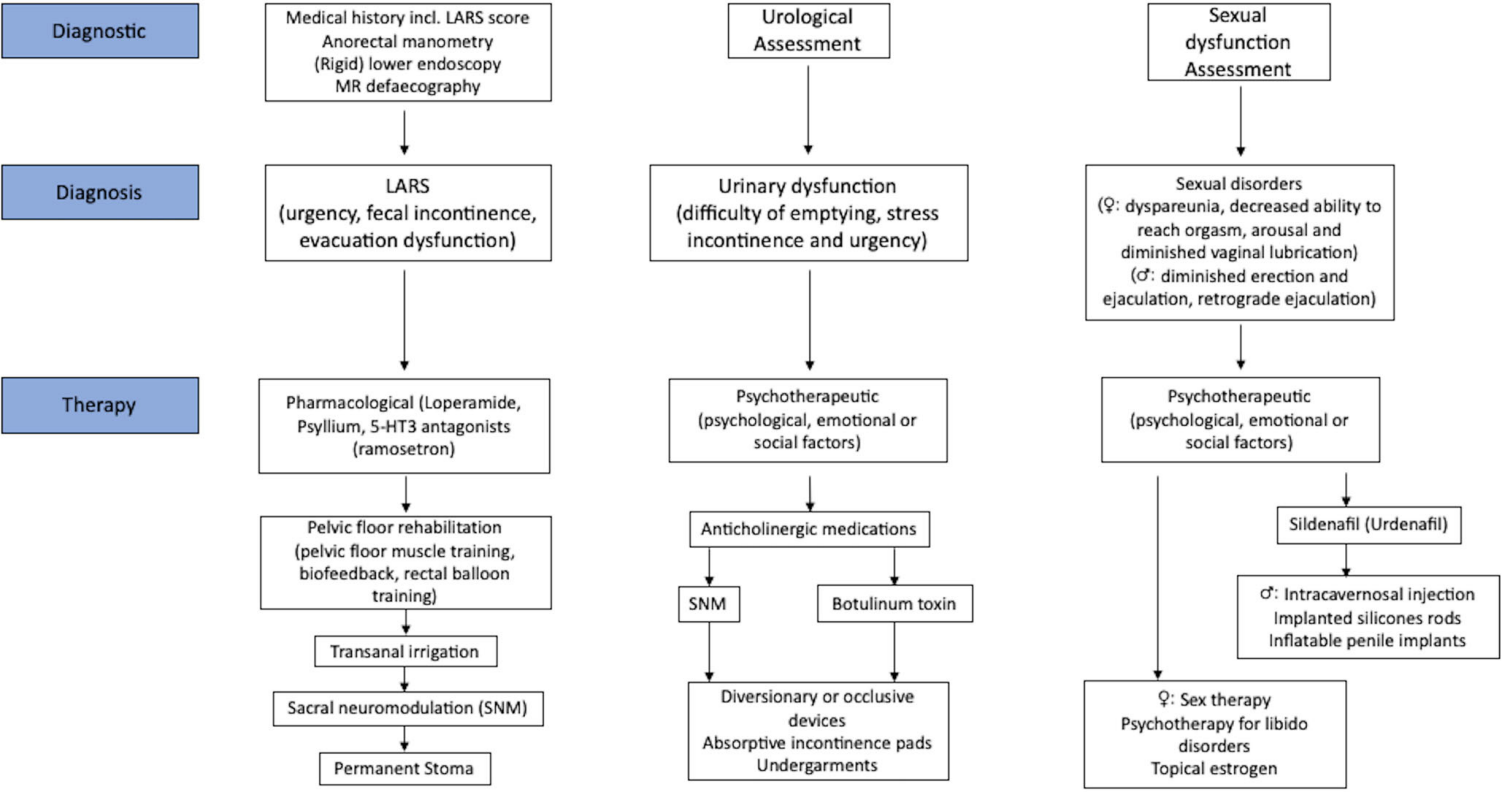
Diagnostic and therapeutic algorithm for low anterior resection syndrome (LARS) and urogenital dysfunctions after anterior resection (AR) for rectal cancer [SNM: sacral neuromodulation]

Concerning the therapy loperamide, psyllium, as well as 5-HT3 antagonists are proven to have some effect on LARS. A cornerstone in therapy of LARS is furthermore PFR including BF. There seems to be evidence for the use of TAI and for SNM to treat incontinence and clustering. In case of bladder dysfunction and sexual dysfunction, patients should be referred early to urology. A proposal of a diagnostic and therapeutic algorithm for dysfunction after AR is depicted in Fig. 2.

LARS as well as urogenital dysfunction after AR, especially LAR, are frequent. Functional disorders after rectal cancer surgery are largely underestimated among health care professionals. Preoperative screening tools to predict LARS are rarely used and information given to patients is often insufficient [207]. Information dedicated to patients and relatives on websites of colorectal clinics about LARS is lacking important content and material is too complex to understand [208]. Patients experience a lack of supportive care after surgery for functional complaints and do not know who to counsel [209]. With regard to a patient’s empowerment surgeons should provide more practical and readily available information on dysfunction after AR and promptly refer patients to their pelvic floor unit for counselling and therapy.
